# Risk Factors Associated With Lymph Node Metastasis and Recurrence in Surgical Cases of pT1 Colorectal Cancer

**DOI:** 10.7759/cureus.76333

**Published:** 2024-12-24

**Authors:** Ben Sasaki, Masahiro Yamada, Yusuke Mishima, Takahito Ohmine, Masaki Tani, Asahi Sato, Kosuke Toda, Takefumi Yazawa, Hidenori Ohe, Kenya Yamanaka

**Affiliations:** 1 Surgery, Shiga General Hospital, Moriyama, JPN

**Keywords:** adjuvant chemotherapy, colorectal cancer, early colon cancer, early rectal cancer, lymph node dissection, lymph node metastasis, pt1, recurrence risk, t1, tumor budding

## Abstract

Objective

This study aims to investigate the risk factors for lymph node metastasis (LNM) and postoperative recurrence in patients undergoing surgery for pT1 colorectal cancer (pT1-CRC).

Materials and methods

We retrospectively analyzed 150 patients who underwent bowel resection with lymph node dissection for pT1-CRC at our department between September 2011 and December 2021. Univariate and multivariate analyses were performed to examine the effects of sex, depth of tumor invasion, venous invasion, lymphatic invasion, tumor budding (BD), and histological type on LNM and recurrence. We analyzed recurrence-free survival (RFS) curves.

Results

LNM was observed in 21 (14.0%) patients. Univariate analysis identified female sex, undifferentiated histological type, positive lymphatic invasion, and tumor budding grade 2/3 (BD2/3) as significant risk factors for LNM, whereas multivariate analysis identified female sex, undifferentiated histological type, and BD2/3 as independent risk factors. No cancer-related deaths were observed during the median observation period of 60.7 months. The five-year RFS rate differed significantly between LNM- and LNM+ patients, at 97.3% and 66.4%, respectively (p=0.0005). BD2/3 was also the significant risk factor for recurrence in the univariate analysis (p<0.0001). In LNM- patients, the five-year RFS was 98.7% for BD1 and 88.2% for BD2/3 (p=0.0014), while in LNM+ patients, it was 100% for BD1 and 37.0% for BD2/3 (p=0.036), with significant differences observed.

Conclusion

In pT1-CRC patients, female sex, undifferentiated histological type, and BD2/3 were the risk factors for LNM. The recurrence rate was higher in patients with LNM than in those without LNM. Regardless of LNM, BD2/3 was the risk factor for the postoperative recurrence of pT1-CRC.

## Introduction

Although the rate of lymph node metastasis (LNM) in patients with pT1 colorectal cancer (pT1-CRC) is low, recurrent metastases can make salvage curative surgery challenging. Therefore, the decision to proceed with surgery after endoscopic treatment should be made carefully.

Currently, no diagnostic method that can reliably predict LNM in pT1-CRC is available. However, the risk of metastasis is assessed to determine whether bowel resection with lymph node dissection should be considered. According to the European Society of Gastrointestinal Endoscopy (ESGE) guidelines, indications for bowel resection with lymph node dissection include positive vascular invasion, submucosal invasion of 1,000 μm or more, positive vertical margins, and poorly differentiated adenocarcinoma in the submucosal invasion area [1]. The Japanese guidelines recommend bowel resection in cases with submucosal invasion of 1,000 μm or more, histological types such as poorly differentiated adenocarcinoma, signet-ring cell carcinoma, mucinous carcinoma, tumor budding (BD), vascular invasion, and positive vertical margins [2].

A project study by the Japanese Society for Cancer of the Colon and Rectum reported a 12.5% rate of LNM in pT1-CRC with submucosal invasion of 1,000 μm or more [3]. Although most patients do not have LNM, salvage surgery may not be feasible if metastasis or recurrence occurs in patients managed with observation alone. Therefore, considering this risk when deciding on a treatment is crucial. Moreover, BD2/3 is recognized as a risk factor for recurrence in stage III colorectal cancer, i.e., colorectal cancer with LNM [4]. However, no studies, including subgroup analyses [4] or other previous publications, have specifically examined the risk of recurrence in stage III colorectal cancer limited to pT1-CRC.

In this study, our objective was to identify the key risk factors for LNM and postoperative recurrence in pT1-CRC by analyzing our surgical cases, including those undergoing additional surgery following endoscopic treatment and those treated with upfront surgery. Furthermore, we aim to use our results to reconsider postoperative surveillance strategies, including the use of adjuvant chemotherapy, for patients at a high risk of recurrence.

## Materials and methods

The study is designed as a single-center historical cohort study. Of 1070 consecutive colorectal cancer surgery cases performed at Shiga General Hospital, Japan, from September 2011 to December 2021, 150 cases were included in the analysis, excluding 14 of 164 cases with pathologically diagnosed T1. The 14 excluded cases included one recurrence at the initial examination, two cases with no confirmed diagnosis of pT1a or pT1b, one case of chemoradiotherapy after endoscopic resection, and 10 cases where BD could not be assessed due to post-treatment at another hospital. About 41 of 150 patients underwent additional resection after endoscopic treatment. In this study, risk factors for LNM and postoperative recurrence were investigated. The candidate risk factors were analyzed as pT1b (submucosal invasion deeper than 1,000 μm), positive vascular invasion, positive lymphatic invasion, tumor growth grade 2/3 (BD2/3), and sex. Regarding BD, to eliminate inter-observer variability, a single experienced pathologist made the diagnosis using hematoxylin-eosin staining.

Statistical analysis was performed using Fisher’s exact test for univariate analysis. Factors that showed significant differences in the univariate analysis were further examined using logistic regression for multivariate analysis. Survival curves were analyzed using the Kaplan-Meier method, and comparisons were made using the log-rank test. Statistical significance was set at p-value < 0.05. For data analysis, we used GraphPad Prism Version® 10.2.0 and JMP® Version 18 software.

Ethical approval for this study was obtained from the Ethics Committee of Shiga General Hospital (No. 231011-01).

## Results

Clinicopathological background

The clinical and pathological characteristics of the 150 patients with pT1-CRC were summarized in Table 1 based on positive and negative LNM.

**Table 1:** Clinicopathological characteristics categorized by the presence or absence of LNM *: median (range); **: "right side" refers to the cecum, ascending colon, and transverse colon, while the "left side" refers to the descending colon through to the anus LNM: lymph node metastasis

Variables		LNM+ (n=21)	LNM- (n=129)
		n (%)	n (%)
Sex	Female	14 (66.7)	39 (30.2)
	Male	7 (33.3)	90 (69.8)
Age*		66 (39-85)	69 (36-88)
Tumor location**	Right side	4 (19.0)	38 (29.5)
	Left side	17 (81.0)	91 (70.5)
Additional resection after endoscopic surgery		6 (28.6)	35 (27.1)
Operative procedure	Laparotomy	2 (9.5)	10 (7.8)
	Laparoscopic surgery	19 (90.5)	119 (92.2)
Lymph node dissection	D1	0 (0)	6 (4.7)
	D2	8 (38.1)	58 (45.0)
	D3	13 (61.9)	65 (50.4)
Number of examined lymph nodes*		13 (5-48)	13 (0-111)
Depth of tumor	pT1a	1 (4.8)	8 (6.2)
	pT1b	20 (95.2)	121 (93.8)
Venous invasion	No	15 (71.4)	103 (79.8)
	Yes	6 (28.6)	26 (20.2)
Lymphatic invasion	No	13 (61.9)	110 (85.2)
	Yes	8 (38.1)	19 (14.8)
Budding	1	14 (66.7)	109 (84.5)
	2/3	7 (33.3)	20 (15.5)
Histology	Differentiated	18 (85.7)	127 (98.4)
	Undifferentiated	3 (14.3)	2 (1.6)

The cohort included 97 males and 53 females, with a median age of 69 years. Tumors tended to be more commonly located in the left-sided colon. Of the total, 41 patients underwent additional resection following endoscopic treatment. Laparoscopic surgery was performed in 138 (92.0%) patients, and D2 or greater lymph node dissection was performed in 144 (96.0%) patients, with a median of 13 lymph nodes examined. Concerning surgical complications, no complications classified as grade 4 and 5 of the Clavien-Dindo classification were observed, but postoperative complications classified as grade 3b were observed in six (4%) patients after laparoscopic surgery. Of these, anastomotic hemorrhage occurred in three (2%) cases, and anastomotic leakage, deep vein thrombosis, and acute gastric ulcer bleeding occurred in one (0.7%) case each.

Regarding the pathological background, 9 patients with pT1a-CRC and 141 patients with pT1b-CRC were reported. LNM was observed in 21 (14.0%) patients. BD2/3 was identified in 27 (18.0%) patients and undifferentiated carcinoma in 5 (3.3%) patients.

Associated factors for lymph node metastasis

**Figure 1:**
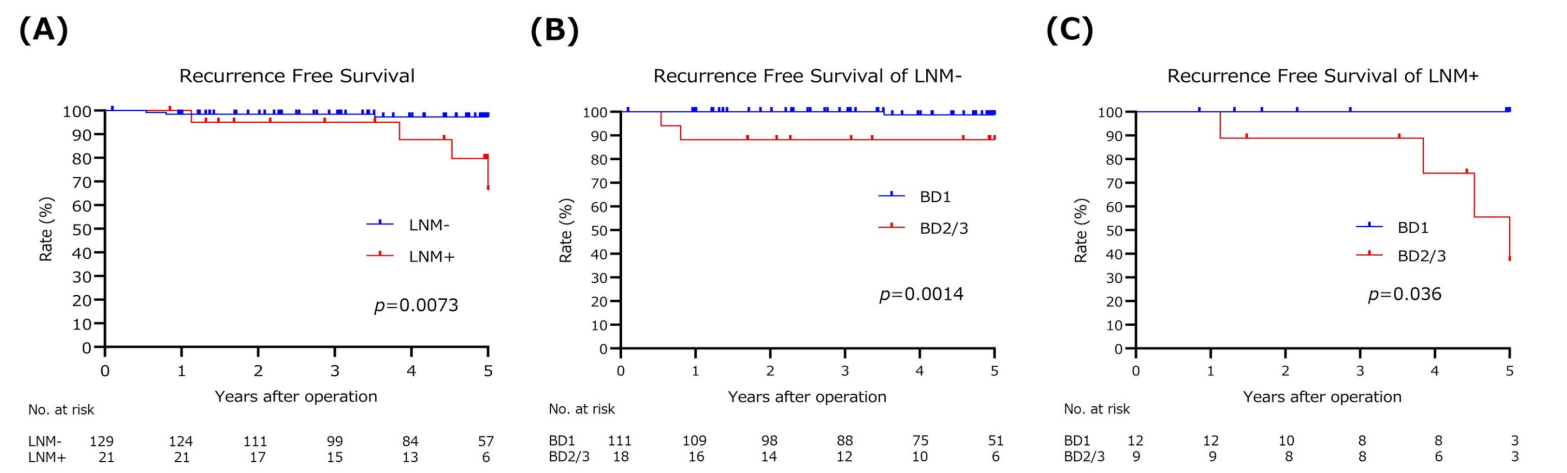
Recurrence-free survival curves. (A) All patients by LNM, (B) LNM- patients by BD status, and (C) LNM+ patients by BD status The log-rank test was used to compare the two groups in the Kaplan-Meier analysis, with a p-value of <0.05 considered statistically significant. LNM; lymph node metastasis, BD; budding

The five-year recurrence-free survival (RFS) rate was 97.3% in LNM- cases and 66.4% in LNM+ cases (p=0.0005), indicating a significant difference based on the presence of LNM (Figure 1A).

Therefore, as shown in Table 2, we investigated the factors associated with LNM in patients with pT1-CRC.

**Table 2:** Factors associated with LNM Univariate analysis for each factor was performed using Fisher's exact test, and multivariate analysis was conducted using logistic regression. A p-value of <0.05 (*) was considered statistically significant. LNM; lymph node metastasis; BD: tumor budding

Variables	Reference	LNM+ (n=21)	Univariate analysis		Multivariate analysis		
	Test	n (%)	Odd Ratio	p-value	Odd Ratio	95%CI	p-value
Sex	Male	7 (7.2)	4.61	0.002*	6.48	2.19-22.1	0.0006*
	Female	14 (26.4)					
pT	1a	1 (8.3)	1.86	0.530	-	-	-
	1b	20 (14.5)					
Venous invasion	No	15 (12.7)	1.58	0.397	-	-	-
	Yes	6 (18.8)					
Lymphatic invasion	No	13 (10.6)	3.56	0.017*	3.23	0.97-10.5	0.056
	Yes	8 (29.6)					
Budding	BD1	12 (9.8)	4.63	0.004*	6.06	1.88-20.5	0.003*
	BD2/3	9 (33.3)					
Histology	Differentiated	18 (12.4)	10.6	0.014*	13.1	1.69-130.7	0.015*
	Undifferentiated	3 (60.0)					

Univariate analysis of risk factors for LNM revealed significant risk factors, including female sex (OR 4.62, p=0.002), lymphatic invasion (OR 3.56, p=0.017), BD2/3 (OR 4.63, p=0.004), and undifferentiated histological type (OR 10.6, p=0.014). In multivariate analysis, female sex (OR 6.48, 95% CI: 2.19-22.1, p=0.0006), BD2/3 (OR 6.06, 95% CI: 1.88-20.5, p=0.0028), and undifferentiated histological type (OR 13.1, 95% CI: 1.68-130.7, p=0.015) were identified as independent risk factors.

Postoperative recurrence

The median follow-up period in our cases was 60.7 months. No cancer-related deaths were observed during this period. Table 3 summarizes the recurrence status. Among the 21 LNM+ patients, 15 received adjuvant chemotherapy.

**Table 3:** Distribution of recurrence stratified by LNM, BD status, and adjuvant chemotherapy LNM: lymph node metastasis; BD: tumor budding

Variables		Recurrence		Total
		- (n=143)	+ (n=7)	
LNM	No	126	3	129
	Yes	17	4	21
Budding	BD1	122	1	123
	BD2/3	21	6	27
Adjuvant chemotherapy	No	131	4	135
	Yes	12	3	15

A total of seven (4.7%) patients experienced recurrence, of whom four were LNM+ and three were LNM-. Of the seven patients of recurrence, three LNM- patients did not receive adjuvant chemotherapy as recommended by the guidelines, while among the four LNM+ patients, one did not receive adjuvant chemotherapy, and three received fluoropyrimidine-based adjuvant chemotherapy without oxaliplatin. Regarding recurrence sites, among the three recurrences in LNM- cases, two were BD2/3, and both recurred as liver metastases. The remaining case involved recurrence at the anastomosis site. R0 resection was achieved in all patients after reoperation. Among the four recurrences in the LNM+ cases, all were BD2/3, with two cases of lung metastasis, one case of lateral LNM, and one case of para-aortic LNM. Univariate analysis shows that LNM+ (OR 9.88, p<0.0001) and BD2/3 (OR 31.7, p<0.0001) were the significant risk factors for recurrence (Table 4).

**Table 4:** Factors associated with recurrence Univariate analysis for each factor was performed using Fisher's exact test. A p-value of <0.05 (*) was considered statistically significant. LNM: lymph node metastasis; BD: tumor budding

Variables	Reference	Recurrence (n=7)	Univariate analysis	
	Test	n (%)	Odd ratio	p-value
Sex	Male	4 (4.1)	1.40	0.70
	Female	3 (5.7)		
pT	1a	0 (0)	-	1.00
	1b	7 (5.1)		
Venous invasion	No	4 (3.4)	2.95	0.17
	Yes	3 (9.4)		
Lymphatic invasion	No	6 (4.9)	0.75	1.00
	Yes	1 (3.7)		
Budding	BD1	1 (0.8)	34.9	<0.0001*
	BD2/3	6 (22.2)		
Histology	Differentiated	7 (4.8)	-	1.00
	Undifferentiated	0 (0)		
LNM	No	3 (2.3)	9.88	0.008*
	Yes	4 (19.0)		

To investigate the association between LNM and BD2/3, we analyzed the survival curves by BD status separately for LNM- and LNM+, which were shown in Figures 1B-1C. These results suggested that BD2/3 might be the risk of recurrence of pT1-CRC, regardless of LNM.

## Discussion

The overall recurrence rate after endoscopic resection of T1-CRC is relatively low, with recent studies reporting a range of 3.1% to 4.7% [5,6], which is consistent with our result of 4.7%. Regarding the pattern of recurrence, extra-regional LNM was observed in half of the LNM+ cases but not in the recurrence of LNM- cases.

LNM worsened in our cases, as well as the prognosis of pT1-CRC, as previously reported [7]. Hematogenous metastasis was the most common recurrence pattern. However, in half of the recurrent cases in LNM+ patients, metastasis occurred in extra-regional lymph nodes. Postoperative adjuvant chemotherapy may be effective in preventing recurrence. Beginning with the Multicenter International Study of Oxaliplatin/Fluorouracil/Leucovorin in the Adjuvant Treatment of Colon Cancer (MOSAIC) trial [8], which randomly demonstrated the additional effect of oxaliplatin, a systematic review reveals that oxaliplatin-containing regimens improved disease-free and overall survival compared to non-oxaliplatin regimens [9]. Of 21 patients with LNM+ (pStage IIIa), 15 (71.4%) received adjuvant chemotherapy after surgery. Of the 15 patients, 4 received adjuvant chemotherapy with oxaliplatin, and none of them experienced recurrence. In contrast, among the four LNM+ patients who experienced recurrence, three received adjuvant chemotherapy without oxaliplatin, and one did not receive chemotherapy. These findings suggest, consistent with previous reports, the potential efficacy of oxaliplatin-containing adjuvant chemotherapy for LNM+ patients. Among the three recurrent LNM- patients, two were classified as BD2/3. Given that LNM- (pStage I) patients are not eligible for adjuvant chemotherapy under current guidelines, it might be worth considering adjuvant chemotherapy for BD2/3 even in LNM- cases.

Among associated factors for LNM, various reports on the risk factors for LNM have been published. Ueno et al. reported that tumor differentiation, vascular invasion, and budding are risk factors for LNM in pT1-CRC [10]. These findings are reflected in the Colorectal Cancer Treatment Guidelines (2024 edition). Significant predictors of LNM include lymphatic invasion, venous invasion, and poor differentiation [5]. BD has also emerged as a significant risk factor for LNM, demonstrating a high sensitivity and negative predictive value [11,12]. A meta-analysis found that BD-positive pT1-CRC cases had a significantly higher risk of nodal metastasis than budding-negative cases [13]. Deep submucosal invasion (≥1 mm) is another important predictor of LNM [14], but in our study, the distance of invasion into the submucosa was not a significant risk factor for LNM. Yoshii et al. reported that patients with pT1-CRC with deep submucosal invasion as the only risk factor have a very low risk of LNM; however, if other LNM risk factors are present, additional bowel resection offers a better prognosis [15]. Other studies have also identified female sex as a significant risk factor for LNM, with a meta-analysis showing an increased risk of LNM in females [16,17]. These findings are consistent with the results of our analysis. The presence of multiple risk factors increases the likelihood of LNM, with rates ranging from 6.0% for one factor to 100% for four factors [18].

BD is a significant risk factor for postoperative recurrence of pT1-CRC and is strongly associated with LNM, disease recurrence, and cancer-related death at five years [19]. In our cases, BD2/3 was the only significant risk factor for recurrence, in addition to LNM. No studies have identified BD2/3 as a risk factor for recurrence, particularly in cases limited to pT1-CRC. Evaluation of BD in colorectal cancer has been shown to be challenging, particularly for less experienced pathologists [20]. Even with standardized protocols such as the International Tumor Budding Consensus Conference (ITBCC) system, the interobserver agreement remains suboptimal [21]. To address this, automated image processing techniques have been developed, demonstrating the potential to reliably quantify B [22]. However, despite these efforts, the evaluation of BD remains difficult, and further improvements in classification systems and assessment methods are necessary to enhance consistency and prognostic value. Therefore, in this study, we retrospectively reassessed BD by a single experienced pathologist to reduce diagnostic variability among pathologists.

While accurate diagnosis of metastasis and recurrence using ctDNA is highly expected [23], we believe that combining the results of this study will contribute to the future treatment of pT1-CRC, particularly in surgical treatment, postoperative adjuvant chemotherapy, and postoperative surveillance.

Due to the small sample size and the single-center, retrospective nature of the study, our findings cannot be regarded as definitive. Further studies involving larger cohorts are necessary to validate our results and provide a more robust conclusion.

## Conclusions

In pT1-CRC patients, the recurrence rate of LNM+ patients was higher than LNM- patients. Female sex, undifferentiated histological type, and BD2/3 were the risk factors for LNM. Additionally, regardless of LNM status, BD2/3 was the significant risk factor for the recurrence of pT1-CRC patients. Our analysis might be beneficial for guiding additional surgery after endoscopic resection, postoperative multidisciplinary therapy, and surveillance of pT1-CRC patients.
